# Identification of Transcriptome SNPs for Assessing Allele-Specific Gene Expression in a Super-Hybrid Rice Xieyou9308

**DOI:** 10.1371/journal.pone.0060668

**Published:** 2013-04-17

**Authors:** Rongrong Zhai, Yue Feng, Xiaodeng Zhan, Xihong Shen, Weiming Wu, Ping Yu, Yingxin Zhang, Daibo Chen, Huimin Wang, Zechuan Lin, Liyong Cao, Shihua Cheng

**Affiliations:** 1 State Key Laboratory of Rice Biology, China National Rice Research Institute, Hangzhou, Zhejiang, China; 2 College of Agronomy, Shenyang Agricultural University, Shenyang, Liaoning, China; Auburn University, United States of America

## Abstract

Hybridization, a common process in nature, can give rise to a vast reservoir of allelic variants. Combination of these allelic variants may result in novel patterns of gene action and is thought to contribute to heterosis. In this study, we analyzed genome-wide allele-specific gene expression (ASGE) in the super-hybrid rice variety Xieyou9308 using RNA sequencing technology (RNA-Seq). We identified 9325 reliable single nucleotide polymorphisms (SNPs) distributed throughout the genome. Nearly 68% of the identified polymorphisms were CT and GA SNPs between R9308 and Xieqingzao B, suggesting the existence of DNA methylation, a heritable epigenetic mark, in the parents and their F_1_ hybrid. Of 2793 identified transcripts with consistent allelic biases, only 480 (17%) showed significant allelic biases during tillering and/or heading stages, implying that *trans* effects may mediate most transcriptional differences in hybrid offspring. Approximately 67% and 62% of the 480 transcripts showed R9308 allelic expression biases at tillering and heading stages, respectively. Transcripts with higher levels of gene expression in R9308 also exhibited R9308 allelic biases in the hybrid. In addition, 125 transcripts were identified with significant allelic expression biases at both stages, of which 74% showed R9308 allelic expression biases. R9308 alleles may tend to preserve their characteristic states of activity in the hybrid and may play important roles in hybrid vigor at both stages. The allelic expression of 355 transcripts was highly stage-specific, with divergent allelic expression patterns observed at different developmental stages. Many transcripts associated with stress resistance were differently regulated in the F_1_ hybrid. The results of this study may provide valuable insights into molecular mechanisms of heterosis.

## Introduction

Hybridization, a common process in nature, is an important component of plant breeding. This process may lead to speciation, adaptive evolution, and ecological innovation [1], [2], [3], [4], and can give rise to a vast reservoir of allelic variants [5]. Combination of these allelic variants may result in novel patterns of gene action, and is thought to contribute to heterosis, a phenomenon in which hybrids show improved and superior performance compared with either inbred parental line [6], [7], [8]. Most efforts to understand the genetic mechanisms of heterosis have been focused on total gene expression levels in hybrids and their parents, with differential regulation of parental alleles in hybrids not well characterized. Although allele-specific gene expression (ASGE) in inter-specific hybrids has been reported in insects [9], fish [10], mammals [11], [12], and plants [5], [13], [14], illuminating the direct impact of parental alleles on gene regulation, these studies were conducted using a limited number of genes.

Recently-developed next-generation high-throughput RNA sequencing technology (RNA-Seq) has enabled the analysis of genome-wide ASGE, facilitating the examination of allelic contributions to gene expression in hybrids. Allelic expression bias in hybrids has been found to be correlated with parental differences [15], with *trans* effects possibly mediating most hybrid transcriptional differences [16]. No attempt has been made, however, to compare ASGE at different developmental stages on a global transcriptomic scale.

In this study, we focused our research on the late-stage high-vigor super-hybrid rice variety, Xieyou9308, which has a grain yield as high as 12.23×10^3^ kg⋅hm^−2^ and was designated as a ‘super rice’ by the Chinese Ministry of Agriculture in 2005 [17]. Xieyou9308 is derived from a cross between the restorer line R9308 (with 25% *japonica* genetic composition) and the maternal line Xieqingzao B (*indica*). We applied RNA-Seq technology to assess genome-wide ASGE in the hybrid genetic background at tillering and heading stages. Results from this study indicate that the parental alleles have unequivalent functions in the hybrid, which may have an impact on heterosis.

## Materials and Methods

### Plant materials and RNA isolation

Experiments were conducted in 2011 on Xieyou9308, a super-hybrid rice commonly planted in China, and its parents Xieqingzao B (female) and R9308 (male). After approximately 30 d of growth in the field at the China National Rice Research Institute, Fuyang, China, 40 seedlings of each genotype were transplanted into plots of plastic foam floating in a pool filled with nutrient solution. Two roots of each genotype were collected at tillering and heading stages and immediately frozen in liquid nitrogen. Total RNA was extracted from roots with Trizol reagent (Invitrogen, Carlsbad, CA) and purified using an Oligotex mRNA Midi kit (Qiagen, Valencia, CA). RNA quality was assessed on a Bioanalyzer 2100 (Aligent, Santa Clara, CA); all samples were found to have RNA Integrity Number (RIN) values greater than 8.5.

### RNA-Seq library preparation and sequencing

Poly(-A)-containing mRNA was isolated from total RNA in two rounds of purification using poly-T oligo-attached magnetic beads. Purified mRNA was then fragmented using an RNA fragmentation kit, converted to cDNA using reverse transcriptase and random primers, and PCR amplified for 18 cycles (Illumina). PCR products were loaded onto an Illumina Hiseq2000 instrument and subjected to paired-end (100 bp ×2) sequencing for 100 cycles. Processing of fluorescent images into sequences, base calling, and quality value calculations were performed via the Illumina data processing pipeline (version 1.8).

### Single nucleotide polymorphism (SNP) diversity analysis

After filtering out low-quality reads (i.e., reads in which more than 30% bases had Q-scores below 20) from the raw reads, we discarded low-quality bases (Q<20) from the 5′ and 3′ ends of the remaining high-quality reads. Cleaned RNA-Seq reads were mapped to the Nipponbare reference genome (IRGSP build 5.0) using RSEM (v1.1.11) [18]. SNPs were identified by comparing sequencing reads of homologous genes between R9308 and Xieqingzao B according to the following criteria: 1) a SNP was called when all R9308 and/or Xieqingzao B reads produced a consensus base and the base is different from each other. 2) at least 10 reads were needed to support each SNP in each parent. For F_1_ hybrid read counting, transcripts showing less than 20 SNP supporting reads were discarded to increase the power of allele-specific expression data. 3) SNPs occurring at both tillering and heading stages and those with quality scores ≥20 in the output file were retained for further analysis. Allelic bias in hybrids was identified by determining for each SNP if there was significant deviation from the binomial distribution of parental alleles (i.e., the allele ratio in the hybrids deviated from 1.0). The data discussed in this publication have been deposited to GeneBank GEO database under accession number GSE43727 (http://www.ncbi.nlm.nih.gov/geo/query/acc.cgi?acc= GSE43727).

### Transcriptome profile analysis

Differential expression was estimated and tested using the R software module edgeR (R version: 2.14; edgeR version: 2.3.52) [19]. We characterized gene expression levels in terms of reads per kb per million reads (RPKM) [20], caculated false discovery rate (FDR) for each transcript, and estimated fold changes (FC) and log_2_ values of FC. Transcripts that exhibited an FDR ≤0.05 and an estimated absolute log_2_ (FC) ≥1 were considered to be significantly differentially expressed. Transcript coverage was estimated as the number of mapped reads for a locus multiplied by 100 bp and then divided by the summed exon length of that locus.

## Results

### Deep sequencing and mapping of RNA-Seq reads

**Figure 1:**
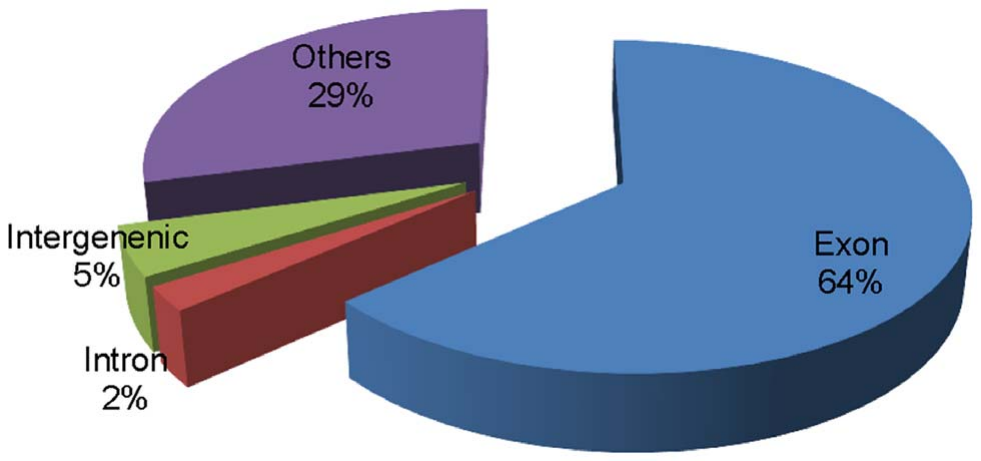
Distribution of reads from RNA-Seq.

RNA-Seq technology is a powerful approach for transcriptional analysis and ASGE assessment [21], [22]. To measure ASGE patterns in rice, we amplified cDNA fragments from a heterotic cross involving Xieyou9308, its maternal line Xieqingzao B, and paternal line R9308, and sequenced them on an Illumina Hiseq2000 platform. In total, 448 million short reads were obtained at tillering and heading stages, with 391 million high-quality 100-bp reads selected for further analysis. With respect to gene expression levels, the two biological replicates were in good agreement (0.86< *R* ^2^<0.96). We then pooled and aligned the short reads against the Nipponbare reference genome (IRGSP build 5.0), and found that 50.32–73.09% of reads were mapped to exonic regions, 2.12–2.83% to intronic regions, and 4.04–5.66% to intergenic regions (Figure 1). A total of 38,872 annotated transcripts were represented by at least one sequence read. On average, 9301 reads were detected per identified annotated transcript, providing greater than 70-fold coverage of the annotated transcriptome.

### Identification of SNPs

**Figure 2:**
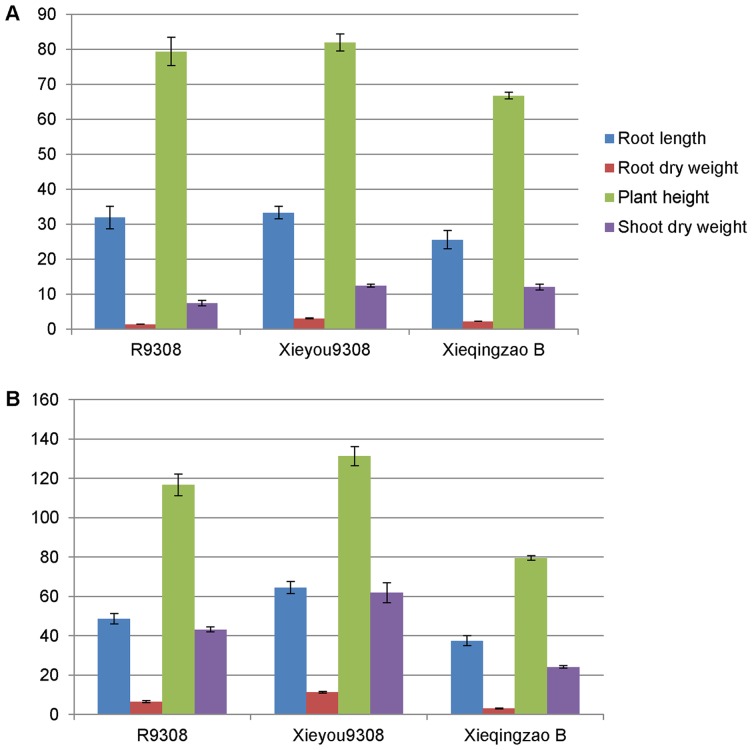
Illustration of heterosis in Xieyou9308 at tillering (A) and heading (B) stages.

**Figure 3:**
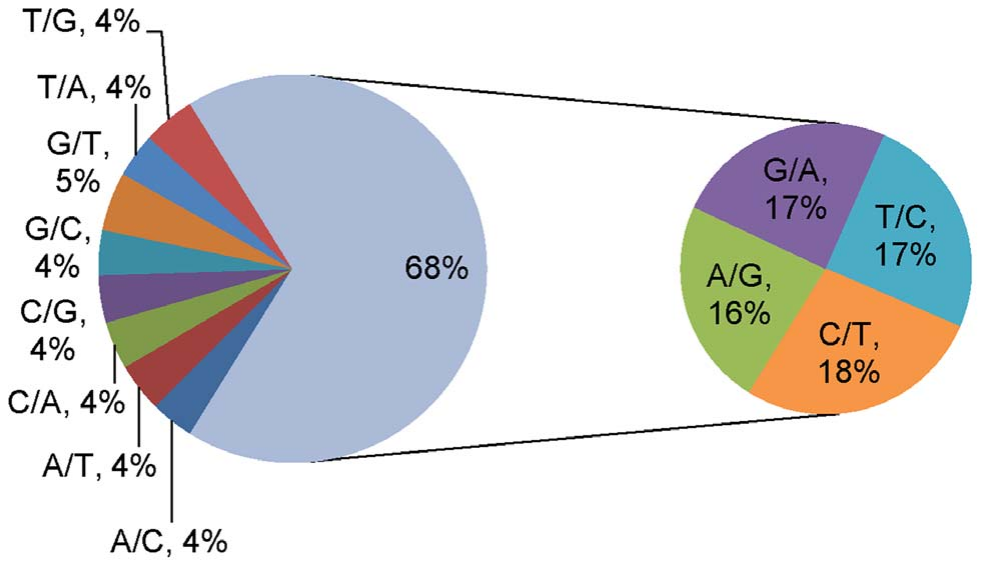
Relative proportion of SNP types in R9308 versus Xieqingzao B.

Xieyou9308 is an elite super-hybrid rice processing superior root and aboveground phenotypes relative to its two parents (Figure 2). Although this superior performance may be due to interaction of the two genomes, the genetic mechanisms involved in producing such hybrid phenotypes are not well understood. To investigate ASGE in the hybrid, we identified SNPs from sequencing reads by comparing each base position in exons of 38,872 annotated transcripts. After applying quality control criteria, 9325 SNPs (3746 transcripts) were further analyzed (Table S1). The most frequently occurring SNPs involved C to T substitutions or their complements (corresponding to G to A mutations on the opposite strand). Combined, these mutation types accounted for 68% of all SNPs detected between R9308 and Xieqingzao B (Figure 3); this percentage is twice that expected if all types of mutations were equally likely. Because the genome of R9308 and Xieqingzao B has been sequenced in 2010, we selected a subset of 25 SNPs and compared them from genome sequencing and RNA-Seq. The results showed that the SNPs from the two methods were in good agreement.

### Allelic bias of gene expression is correlated with parental differences

**Figure 4:**
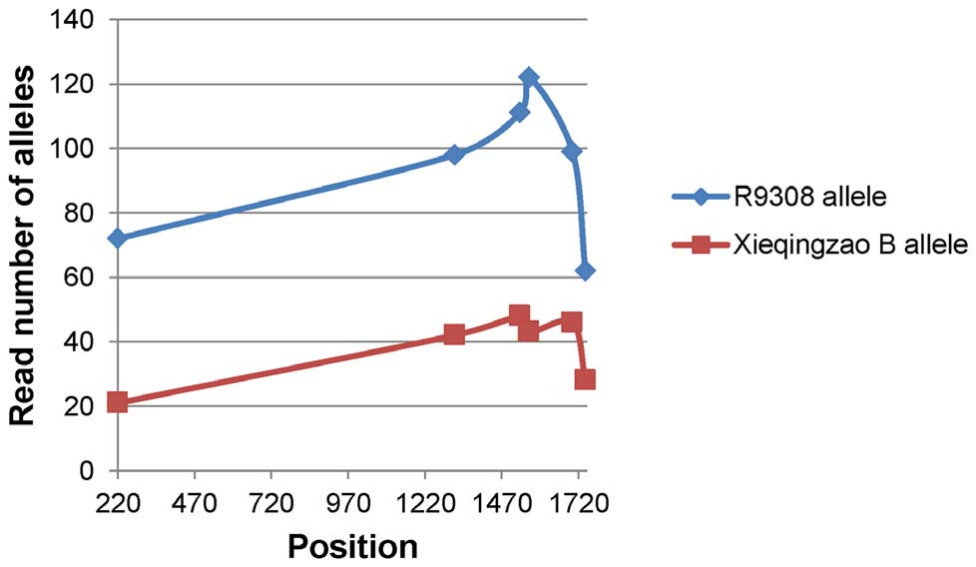
Example of a transcript showing allelic bias in Xieyou9308 at heading stage. Number of reads detected for a given parental allele at each SNP position is plotted.

**Figure 5:**
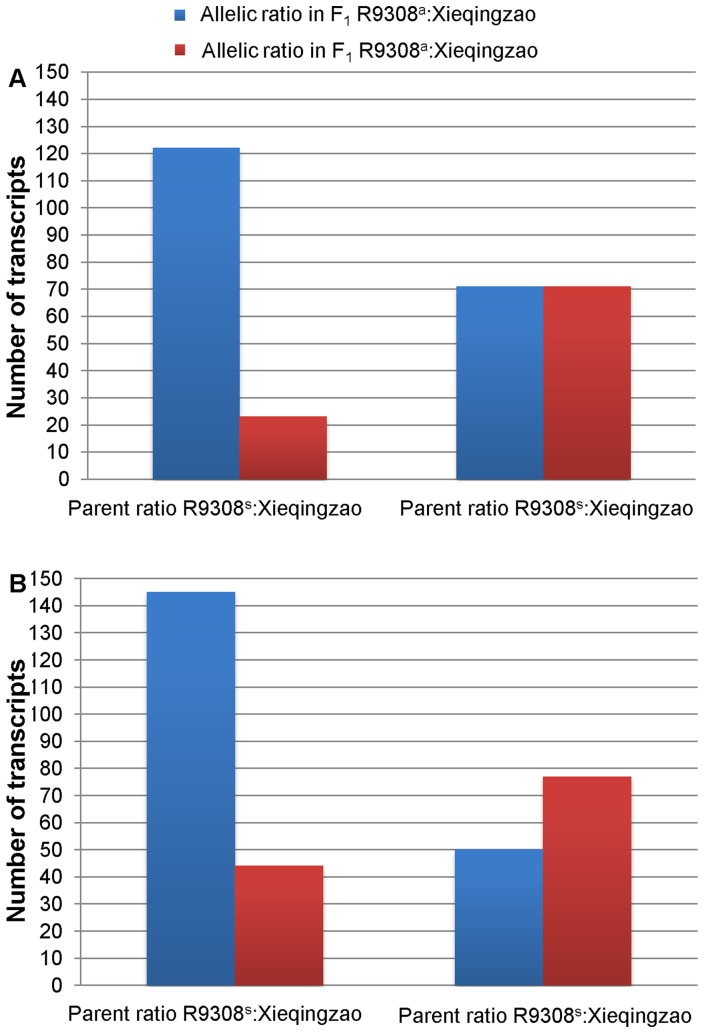
Allelic biases in Xieyou9308 according to their differences between parents. R9308^s^, gene expression levels in R9308; Xieqingzao B^s^, gene expression levels in Xieqingzao B; R9308^a^, R9308 allele; Xieqingzao B^a^, Xieqingzao B allele.

To ensure accuracy and reliability, only SNPs exhibiting a significant allelic bias (*P*<0.01) were included in our analyses. In addition, among those transcripts with more than one SNP identified, two showed a contradictory allelic expression bias for different SNPs and were excluded from further analyses. Out of the 4685 identified SNPs (2793 transcripts) (Table S2), significant allelic biases were exhibited in 336 SNPs (289 transcripts) from the tillering stage and 376 SNPs (316 transcripts) from the heading stage (Figure 4) (Table S3, Table S4). We also investigated the effects of gene expression differences between the parental lines on allele-specific patterns in the hybrid. We designated the ratio of expression between R9308 and Xieqingzao B as R9308^s^/Xieqingzao B^s^ and the ratio of allelic expression in the hybrid Xieyou9308 as R9308^a^/Xieqingzao B^a^. We found that most transcripts with higher levels of gene expression in R9308 also exhibited allelic biases towards R9308 in the hybrid (Figure 5).

### Assessment of ASGE in the F_1_ hybrid

**Figure 6:**
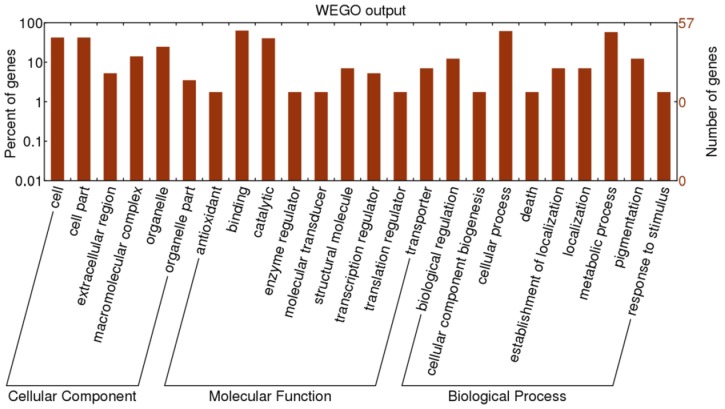
GO classification of 92 R9308-biased transcripts.

We examined allelic expression differences between tillering and heading stages and found that 480 transcripts showed allelic expression biases during at least one stage, with 125 showing allelic expression biases at both tillering and heading stages and 355 showing allelic expression biases at only one stage. Out of the 125 transcripts exhibiting biases at both stages, 92 showed allelic expression biases towards the R9308 allele and 26 towards the Xieqingzao B allele. We further investigated the 92 R9308-biased transcripts using Web Gene Ontology Annotation (WEGO) software [23] and found that these transcripts could be classified into a diversity of functional subcategories, such as cell and cell part in the cellular component category, binding and catalytic processes in the molecular function category, and cellular and metabolic processes in the biological process category (Figure 6). In addition, 195 of 289 transcripts at the tillering stage and 195 of 316 transcripts at the heading stage showed allelic expression biases towards the R9308 allele.

To further investigate transcripts showing strong allelic expression biases in the F_1_ hybrid, we analyzed 38 transcripts exhibiting expression almost exclusively from one parental allele (the fraction of reads carrying R9308 allele was less than 0.3 or greater than 0.7 at tillering or heading stages) (Table S5). Among these 38 allele-specific expressed transcripts, 20 predominantly expressed the R9308 allele at both stages, and 14 primarily expressed the Xieqingzao B allele. In addition, four transcripts showed different allelic biases between the two stages. The annotation indicated that 26 transcripts could be functionally characterized. Transcripts encoding proteins involving resistance to diseases or other stresses, such as nucleotide-binding adaptor shared by APAF-1, R proteins, and CED-4 (NB-ARC) protein, pathogenesis-related (PR) protein, chitin elicitor receptor kinase, plant disease resistance response protein, and nucleotide binding site-leucine rich repeat (NBS-LRR protein), were predominant in the annotated list (Table 1).

**Table 1:** Total and allelic expression of six transcripts at tillering and heading stages.

Transcript ID	R/X12^a^	R/X12^s^	R/X34 a	R/X34 s	Annotation
Os02t0272900	4.500	7.001	3.467	4.852	NB-ARC domain containing protein
Os07t0127700	3.087	3.347	2.444	1.995	Similar to Pathogenesis-related protein class 1
Os07t0129200	2.960	3.670	2.696	0.717	Similar to Pathogenesis-related protein PR1a
Os09t0511000	2.786	1.214	2.667	1.436	Chitin Elicitor Receptor Kinase
Os07t0617100	0.222	0.422	0.298	0.063	Plant disease resistance response protein family protein
Os11t0229300	0.292	0.284	0.267	0.377	Similar to NBS-LRR protein

R/X12: the ratio of R9308 to Xieqingzao B at the tillering stage; R/X34: the ratio of R9308 to Xieqingzao B at the heading stage.

a Allele-specific gene expression in the hybrid.

s Total gene expression in parents.

We further analyzed total and allelic expression with respect to the two parents and the hybrid in the above-mentioned resistance transcripts. Two transcripts (Os02t0272900 and Os11t0229300) at both tillering and heading stages and one transcript (Os07t0617100) at the heading stage showed significant total expression differences between parental strains. The other transcripts did not show significantly different total expression between parental strains. For Os11t0229300, R9308^a^/Xieqingzao B^a^ ratios in the hybrid were as expected from R9308^s^/Xieqingzao B^s^ ratios of the parental strains; however, R9308^a^/Xieqingzao B^a^ ratios of Os02t0272900 in the hybrid were lower than expected from R9308^s^/Xieqingzao B^s^ ratios.

## Discussion

ASGE in hybrids can be studied using two general approaches [24]. The first approach, a polymorphism-directed method, utilizes known genome variants and can achieve highly ASGE results [9], [25]. A second approach employs SNP arrays to examine tens of thousands of ASGE sites simultaneously [13], [26]. Both approaches, however, requires prior knowledge of genomic information. Another approach, used in this study, is based on RNA-Seq, does not rely on previous knowledge of genetic variation and provides an unbiased view of gene regulation. The single-base resolution obtained using this method provides information regarding both transcript abundance and allelic bias. In this study, we employed Illumina/Solexa sequencing to identify SNPs between the parental lines R9308 and Xieqingzao B and quantify ASGE in the F_1_ hybrid Xieyou9308.

Interestingly, we found that CT and GA SNPs between R9308 and Xieqingzao B constituted nearly 68% of all identified SNPs, consistent with a previous study in which these SNP types accounted for 73% of SNPs between Nipponbare and 93–11 [16]. This phenomenon may be due to methylated cytosines that mutate more frequently than non-methylated cytosines. In addition, deamination of methylcytosine, yielding thymine, occurs at higher rates than other spontaneous mutations [16], [27], [28], [29]. DNA methylation is a heritable epigenetic mark; it can control gene expression and environmental stress responses, and may play a role in heterosis in plants [16], [30], [31]. In a future study, we plan to determine if DNA methylation is present in the parents and their F_1_ hybrid, and, if so, to characterize how methylation from inbred parents interacts during generation of their hybrid progeny and contributes to heterosis.

In our study, only 480 (17%) of 2793 identified transcripts showed significant allelic biases at tillering and heading stages. A similar result was observed in another recent study, in which 398 (22.7%) out of 1754 genes exhibited significant allelic expression differences in a reciprocal F_1_ hybrid between Nipponbare and 93–11 [15]. Differences in allelic expression were also observed in 73% and 57% of identified genes in maize and *Populus*, respectively [5], [14]. In contrast, in a study of hybrid mice, allelic expression differences were found in only 10% of genes [11]. The relatively high allelic expression variation observed in maize and *Populus* is probably a consequence of their highly polymorphic genomes. Similarly, the genetic divergence between *indica* and *japonica* subspecies may account for the higher degree of allelic expression variation observed compared with mouse hybrids [5].

Of the 480 transcripts that showed allelic expression biases during at least one stage, 67% and 62% were biased towards the R9308 allele at tillering and heading stages, respectively. In addition, transcripts with higher gene expression levels in R9308 also exhibited R9308 allelic biases in the hybrid. Furthermore, 92 (74%) of the 125 transcripts exhibiting allelic expression biases at both tillering and heading stages were biased towards the R9308 allele at both stages and were associated with different functional proteins. These results indicate that R9308 alleles tend to preserve their characteristic activity states in the hybrid and may play important roles in hybrid vigor at both stages. Functional diversity of the R9308 alleles in the hybrid may have an impact on hybrid performance. Interestingly, about 355 (74%) of the 480 transcripts with allelic biases showed divergent allelic expression patterns at different developmental stages, indicating that allelic expression in hybrids can be highly stage-specific [5], [32].

Allelic variation in gene expression may arise from *cis*- and/or *trans*-regulatory elements [9]. Alteration of *cis*-elements may affect aspects such as promoter strength, enhancer action, or transcript stability, whereas *trans*-element changes may involve structure, binding affinities, or intercellular levels of factors inﬂuencing transcription [21]. *Cis*- and *trans*-regulation can be distinguished by comparing ratios of species-specific transcripts between F_1_ hybrids and parental lines [5]. If a mutation occurs in a *cis*-element, the affected gene shows the same ratio of allelic expression levels in both the hybrid and parents. On the other hand, if *trans*-elements are altered in the parents, no differences in allelic expression are displayed for that gene in the F_1_ hybrid because both alleles are exposed to the same subcellular environment. In our study, most transcripts (89.65% at the tillering stage and 88.69% at the heading stage) exhibited relatively balanced allelic expression in the hybrid genetic background, suggesting that *trans* effects may dominate genetically mediated allele-specific expression [18].

Based on findings of a previous study, alleles derived from different parents may be differentially regulated in hybrids during plant development and in response to environmental signals [14]. In our study, we analyzed 38 transcripts exhibiting expression biases towards one parental allele. Four transcripts encoding UPF0307 protein, ATP-binding cassette (ABC) transporter protein, and Zinc finger RING-type domain containing protein displayed different allelic biases between the two stages, suggesting differential roles for the alleles during hybrid development. A majority of the 38 transcripts, however, showed consistent allelic biases and were associated with different functional proteins. It should be noted that these transcripts were relatively enriched in pathways for resistance to diseases or other stresses, implying that this category may be involved in vigorous growth in the hybrid and deserves further investigation. Furthermore, the R9308^a^/Xieqingzao B^a^ ratios of Os11t0229300 (encoding the NBS-LRR protein) was as expected from R9308^s^/Xieqingzao B^s^ ratios, implying the involvement of *cis*-regulation; in contrast, R9308^a^/Xieqingzao B^a^ ratios of Os02t0272900 (encoding the NB-ARC protein) were lower than expected R9308^s^/Xieqingzao B^s^ ratios, suggesting both *cis-* and *trans*-regulation. The differential regulation of the two parental alleles of these resistant transcripts may contribute to Xieyou9308 hybrid vigor.

## Conclusions

In this study, roots from tillering and heading stages of the super-hybrid rice Xieyou9308 and its parents were used for global transcriptional analysis and assessment of ASGE. Our results demonstrate that Illumina paired-end sequencing is a powerful tool for exploring allelic expression patterns and gene regulatory phenomena in interspecies hybridization, and may provide valuable information to further elucidate molecular mechanisms of heterosis.

## Supporting Information

Table S1 **SNPs detected in the hybrid at tillering and heading stages.**
(XLSX)

Table S2 **SNPs showing consistent allelic expression biases in the hybrid.**
(XLSX)

Table S3 **SNPs showing significant allelic biases for accumulated transcripts in the hybrid at the tillering stage.**
(XLSX)

Table S4 **SNPs showing significant allelic biases for accumulated transcripts in the hybrid at the heading stage.**
(XLSX)

Table S5 **Thirty-eight transcripts showing biases towards one parental allele in the F_1_ hybrid.**
(XLSX)
